# 助教制分组轮转式实验教学模式的改革与探索——以药物分析实验课为例

**DOI:** 10.3724/SP.J.1123.2024.03012

**Published:** 2025-02-08

**Authors:** Ran ZHAO, Ling ZHANG, Kun ZHANG, Youxin LI

**Affiliations:** 天津大学医学部药物科学与技术学院,天津300072; School of Pharmaceutical Science and Technology, Faculty of Medicine, Tianjin University, Tianjin 300072, China

**Keywords:** 药物分析, 仪器分析, 轮转, 助教, 教学改革, pharmaceutical analysis, instrumental analysis, rotation, teaching assistant, teaching reform

## Abstract

药物分析实验课是药学专业人才培养过程中的重要一环,它注重学生理论知识在实际中的应用能力和大型仪器的使用能力,进而启发学生的创新思维,培养学生的应用开发能力。针对目前国内外药物分析实验课存在的不足,如国内高校实验课程设置的内容差异较大,相对滞后,且受经济、人力等条件的制约,学生缺少操作使用大型分析仪器的训练,国外高校开课配置的专业仪器不够全面,实验课时少,供实验教学使用的大型仪器数量有限,天津大学医学部药物科学与技术学院在药物分析实验教学中探索了助教制分组轮转式实验教学模式,依托学院大型仪器共享平台,引入“助教”“轮转”等教学方式,形成了一套较为完善的教学模式。该模式通过延长时间轴的方式降低某一时间点对实验仪器数量的需求,缓解了大型仪器分配的压力,同时按照课程要求系统设计了一套完整的药物分析教学方案,实验内容范围广、深度大,考核方式新颖全面,贴近药学专业行业标准和仪器分析前沿,涵盖了药物分析中常见的分析方法和仪器;借助实验助教分组教学,帮助学生巩固理论知识,系统掌握药物质量控制的检测项目和药物研究流程,并为学生开发新的药物分析方法奠定基础。经过多年的不断尝试与探索,该模式日臻成熟完善,取得了良好的教学效果,期望为国内高校药物分析实验课程的开展提供借鉴。

## Abstract (en)

Experimental courses in pharmaceutical analysis are an important part of the training process for pharmaceutical talent. These courses focus on applying theoretical knowledge in practice and using large instruments, with the aim of inspiring innovative thinking and cultivating student development. Currently, several issues impede the success of experimental pharmaceutical analysis courses both in China and abroad. In domestic colleges and universities, the content of these courses varies significantly and is relatively unsophisticated. Owing to economic and human resource limitations, students lack training in the operation and use of large analytical instruments. In foreign universities, the range of professional instruments is not comprehensive, experimental class hours are limited, and the availability of large instruments for experimental teaching is insufficient. The School of Pharmaceutical Science and Technology, Faculty of Medicine, Tianjin University, explored the teaching assistant system with group rotation for the delivery of experimental pharmaceutical analysis courses. In combination with the large instrument sharing platform of the school, this approach resulted in a relatively comprehensive teaching model. This model reduced the demand for experimental instruments at a certain time by extending the time axis, thereby relieving the pressure on large instrument allocation. Furthermore, a complete pharmaceutical analysis teaching program was designed according to the curriculum requirements, covering common analytical methods and instruments used in pharmaceutical analysis. With guidance from experimental teaching assistants, students consolidated their theoretical knowledge, mastered key techniques for drug quality control and the drug research process, and laid a foundation for developing new drug analysis methods. Over years of continuous exploration, this model has matured to achieve good teaching outcomes. This work can provide a reference for the development of experimental pharmaceutical analysis courses in domestic universities.

药物分析是分析化学的一个重要分支,主要是运用化学、物理学、生物学以及微生物学的方法和技术来研究药物的化学成分、稳定性、生物利用度、临床监测以及中草药有效成分定性定量检测等的一门学科。它与药物化学、药剂学、天然药物化学等学科联系紧密,并作为研究“工具”贯穿其中,具有很强的实践性。随着科技的发展,药物分析实验教学应注重培养学生对理论知识的应用能力、对大型专业仪器的使用能力,进而启发学生的创新思维以及应用开发能力^[1]^。

目前国内许多高校药物分析实验课程仍按照“一对多”的传统模式开展,即“一位老师演示-全班学生观摩-学生提交实验报告”,这样学生使用实验仪器的机会相对不足,学生参与感不强,对实验的了解只浮于表面,无法充分调动学生的主观能动性,课程效果经常难以达到预期目标。

天津大学医学部药物科学与技术学院以药物分析实验课程为例,探索了助教制小组轮转式实验教学模式。研究生在任课老师的指导下作为实验“助教”负责不同的实验项目,本科生以小组的形式在不同助教之间轮转进行药物分析实验。通过延长时间轴的方式降低某一时间点对实验仪器数量的需求,使每位学生都能参与到药物分析实验中,提高实验课程的效率与效果,激发学生对实验课程的学习兴趣和热情,促进学生对药物分析理论知识的理解和实践技能的掌握。

## 1 药物分析本科实验教学的意义

药物分析是为药品的研究、生产、供应以及临床使用提供严格的质量标准和科学分析方法的学科,以保证用药的安全性、有效性和合理性,因此药物分析课程着重围绕药物质量控制问题进行教学,研究化学合成药物或结构明确的天然药物及其制剂的质量问题。药物分析实验教学涉及物理分析方法、化学分析方法、生物分析方法、仪器分析方法等多种分析方法和手段,需要借助熔点仪、旋光仪、红外光谱仪、紫外分光光度计、薄层色谱、高效液相色谱(HPLC)、液相色谱-质谱联用仪等多种仪器来完成。

通过实验教学,学生学习掌握药物的鉴别、杂质的限量检查、药物含量的测定等一系列药物检测流程和方法,了解国内外药品生产质量管理规范、药品质量监督管理的法规与标准体系,学习相关仪器的结构、工作原理、操作使用,手脑结合,巩固并验证理论知识,以达到培养高层次实用型药学人才的目的,并为学生今后从事药物研究、产、质量控制与管理、流通和临床使用等相关领域的工作打下坚实的基础。

## 2 国内外药物分析本科实验教学开展现状

### 2.1 国内现状

国内高校药物分析实验教学通常在学生完成有机化学、分析化学等课程学习的基础上开展,其课时在24~48学时不等,实验数量在8~12范围内,分为演示实验、验证实验、设计实验等。表1中列举了部分国内高校药物分析实验课程的具体内容。

**表1:** 部分国内高校药物分析实验课实验内容

Exp. No.	Peking University^[2]^	Jiangsu Normal University^[3]^	Nanjing Agricultural University^[4]^
1	introduction to experiments in drug analysis	use and maintenance of pH meter (optional)	basic knowledge for drug analysis experiments
2	glucose impurity check	determination of the absorption coefficients of drugs	preparation and calibration of perchloric acid standard solutions
3	drug impurity evaluation	effect of GC carrier gas flow rate and column temperature on separation (optional)	general impurity check for glucose
4	determination of amiodarone content	selection of HPLC conditions (optional)	calibration of iodine standard solutions and determination of vitamin content
5	extraction and titration of procaine hydrochloride injection	inspection of drug impurities	determination of fluorenone-related substances at high and low concentrations using thin layer chromatography
6	determination of atropine sulfate injection	determination of lidocaine hydrochloride content	inspection of drug impurities
7	determination of ethamsylate injection	identification of ibuprofen using ultraviolet spectrophotometry and infrared spectroscopy (optional)	determination of vitamin A content in vitamin AD capsules using three-point correction
8	determination of methyl isoxazole and trimethoprim in compound sulfamethoxazole tablets using dual-wavelength spectrophotometry	analysis of procaine hydrochloride injection	determination of florfenicol content using HPLC
9	determination of the content of compound acetylsalicylic acid tablets	determination of vitamin E capsules using GC	determination of ethanol content in tinctures
10		determination of salicylic acid content in aspirin capsules	
11		determination of penicillin sodium content for drug injection	
12		determination of vitamin B1 content in tablets using differential spectrophotometry	
Total time	48 h	48 h	27 h

Exp.: experiment.

通过对比分析,发现目前国内高校药物分析实验教学存在一些问题。首先是受经济、课时等因素的制约,实验内容不全面。例如一些操作烦琐、耗能耗时的实验无法在教学实验中完整地呈现出来^[5]^,或者因实验设备数量不足,无法满足学生同时开展实验,只能通过PPT讲解或者老师演示展现。许多高校为了解决这一问题,引入了仿真实验、视频动画、线上教学平台等现代信息技术手段^[6]^,但教学效果与学生亲自动手操作有一定差距。其次,实验内容相对滞后。在药物分析教学实验中,使用滴定、紫外分析方法的实验项目占比偏多,学生缺少学习使用大型精密仪器的机会^[7]^。第三,实验内容的关联度较低。一方面基础课程和专业课程的实验教学往往自成体系,缺乏学科间联系^[8]^;另一方面课程内实验项目涉及多种药物及多种检测手段,相互之间没有联系,缺少系统设计和逻辑关系,学生无法从整体上理解和把握某一特定药物的全面质量控制方法。第四,实验教学中存在安全隐患。实验过程中涉及的学生、试剂、仪器众多,存在诸多安全风险,老师一人无法顾及所有学生的实验操作,难免会因学生操作不当造成仪器损坏甚至人员受伤。

### 2.2 国外现状

国外高校十分重视实验教学在大学生培养中所发挥的作用^[9]^,发达国家的高校更是注重将教学与科研、生产相结合,教学体系相对完善^[10]^。澳大利亚^[11]^的一些高校开设了分析化学课程,但不分设理论课与实验课,课程中实验部分的学时数更多,实验内容主要包括滴定实验及简单仪器分析实验,实验深度广度难度较低。德国哥廷根大学和德国亚琛工业大学未设置专门的药物分析实验课,而是在本科生通过化学理论考核后,参与化学综合实验,涉及大型分析仪器检测的内容则由博士研究生助教协助通过送样检测方式获得目标样品测试结果。本科生真正接触大型分析仪器是在加入导师课题组后,根据科研项目需要接触相应仪器。部分美国高校^[12]^,如犹他州立大学、华盛顿州立大学,开设了专门的仪器分析实验课程,涉及的大型仪器较多,实验内容涉及HPLC、气相色谱、红外光谱、质谱、核磁共振等仪器的使用;密歇根州立大学^[13]^还涉及拉曼光谱等其他高校未涉及的内容。东京大学^[12]^在分析化学课程中安排了X射线光谱分析法和能谱分析两部分实验内容。

然而,这些国外高校实验课的缺点也显而易见,国外高校的实验室大多是综合性的,很少区分基础实验室和专业实验室,因此专业仪器的配置不够全面,不少实验室的硬件条件相对差,实验课时少,内容的深度广度也比较浅显,同时供实验教学使用的大型仪器数量不足的问题也比较突出,无法满足学生全面接触和应用各种药学大型实验仪器的需求^[14]^。实验过程中教授参与指导的机会较少,大多依靠个人探索。

## 3 药物分析小组轮转式本科实验教学改革与探索

天津大学医学部药物科学与技术学院开设的药物分析实验课程以阿司匹林、蒿甲醚及其衍生物为实验对象,系统设计了一套完整的药物分析教学方案,实验内容范围广、深度大,贴近药学专业行业标准和仪器分析前沿,涵盖了药物分析中常见的分析方法和仪器,借助实验助教分组教学,可以帮助学生巩固理论知识,系统掌握药物质量控制的检测项目和药物研究流程。

### 3.1 实验内容的设置

药物分析实验课程分为4部分:药物鉴别、杂质检验、含量测定、蛋白质与阿司匹林的相互作用探索,开设12个实验,每个实验4学时,共48学时,历时3周。

第一部分是药物鉴别。在药物分析中,鉴别是指药物真伪鉴别,即标签与内容物是否一致。本部分实验设计了8种样品,利用6种方法,包括熔点测定、旋光度测定、比色法、薄层色谱法、红外光谱法、紫外分光光度法,通过与5种标准品对比,全面判断8种样品真伪,具体内容见表2。为支持学生使用更多手段鉴定和分析药物结构,本实验还提供核磁共振波谱仪和液相色谱-质谱联用仪等精密仪器供学生使用。

**表2:** 药物鉴别实验

Exp. No.	Title	Purposes	Assay method
1	determination of the melting points of aspirin, artemether, and their derivatives	understand the definition of melting point; master the melting point determination method and its application in the field of drug research	melting point method
2	spectrophotometric determination of aspirin, artemether, and their derivatives	master the principle of polarimetric analysis for drug determination	polarimetry
3	identification of aspirin, artemether, and their derivatives using colorimetry	master the method of drug identification using colorimetry	colorimetry
4	identification of aspirin, artemether, and their derivatives using thin layer chromatography	master the method of drug identification using thin layer chromatography	thin layer chromatography
5	identification of aspirin, artemisinin, and their derivatives using infrared spectroscopy	master the principle and method of drug identification using infrared spectroscopy	infrared spectroscopy
6	identification of aspirin, artemisinin, and their derivatives using ultraviolet spectrophotometry	master the principle and method of drug identification using ultraviolet spectrophotometry	ultraviolet spectrophotometry

通过以上实验,要求学生能够通过实验结果鉴定8种药物标签是否正确,同时掌握这6种鉴别方法,为今后独立建立药物鉴别方法奠定基础。

第二部分是药物杂质限量检查。利用比色法、沉淀法、干燥失重法和HPLC法,对3种阿司匹林样品和3种蒿甲醚样品中的有关杂质进行限量检查。特别是有关杂质限量检查中,根据有无有关杂质标准品,使用了外标一点法和自身对照法,检验学生能否利用这两种方法判断杂质是否超标,为今后运用这两种方法限量测定其他药物有关杂质奠定基础,具体见表3。

**表3:** 杂质检验实验

Exp. No.	Title	Purposes	Assay methods
7	determination of impurities in aspirin and artemether	learn methods for impurity determination in drugs	colorimetry, precipitation method, loss-on-drying method
8	determination of aspirin, artemether, and related substances using HPLC	master the principle and method of drug impurity detection using HPLC; become familiar with the working principle and operation of HPLC; understand routine HPLC maintenance	aspirin: HPLC and one-point external standard method; artemether: HPLC and self-comparison method

第三部分是药物含量的测定。本部分实验涉及3种阿司匹林样品和3种蒿甲醚样品的含量测定,具体内容见表4。针对阿司匹林含量测定,设计了滴定法、HPLC法、紫外分光光度法3种方法,要求学生运用自己所学知识分析这3种方法的优劣及局限性。其中,HPLC法采用标准曲线法定量,以拓展学生们对具体定量方法的认识和掌握。对于蒿甲醚HPLC定量法,要求学生结合蒿甲醚结构和实验六紫外分光光度法获得的全波长紫外光谱图解释检测波长的设定依据,培养学生开发药物分析方法的能力。

**表4:** 药物含量测定实验

Exp. No.	Title	Purpose	Quantitative methods
9	determination of aspirin using HPLC	learn the principle of HPLC for drug analysis applications; learn the method of aspirin content determination using HPLC	HPLC and standard curve method
10	determination of aspirin using titration and ultraviolet spectrophotometry	master the titration method for determining aspirin content; master the ultraviolet spectrophotometry method for determining aspirin content; in combination with experiment No. 9, compare the differences, advantages and disadvantages, and limitations of the three methods for determining aspirin content	titration method (from Chinese Pharmacopoeia), ultraviolet spectrophotometry method (self-developed)
11	determination of artemether using HPLC	master the HPLC method for artemether determination; learn about the relationship between detection wavelength and the structure of artemether	HPLC and standard curve method

第四部分为药物研究相关的探索性实验。药物小分子与血清白蛋白之间的相互作用研究对阐明药物在体内的转运、分配及代谢过程、解释蛋白质结构和功能的关系以及开发新药等都有重要意义,实验12为阿司匹林与牛血清白蛋白(BSA)之间的相互作用。学生使用磷酸盐缓冲液配制pH为7.4的BSA溶液,向其中加入一定量的阿司匹林标准溶液,阿司匹林与BSA结合达到平衡后将上清液在选定波长下使用紫外分光光度计测量,计算游离阿司匹林的浓度。学生通过该实验掌握生物样品的预处理方法,了解紫外分光光度法在检测阿司匹林与BSA相互作用中的应用,为今后开展药物研究奠定基础。

### 3.2 助教制实验教学模式

招募具有相关研究背景的研究生担任实验助教,并在课前接受任课教师的系统培训,确保其掌握所辅助的课程内容。在实验课开课前,授课教师向本科生讲解所有实验内容和注意事项。在实验进行中,助教再次向小组成员介绍当天具体的实验内容与仪器使用,同时确保学生实验过程中的安全,监督本组学生按照正规流程操作,正确处理实验室中的突发状况,授课教师在实验过程中循环与各小组学生交流实验中存在的各种问题。实验结束后,助教清点实验试剂和器材,并对实验仪器进行日常管理和维护。

实验课程共设12个实验项目,每个实验配置2位助教;将学生划分成12个小组,每组4~6人;实验分12天进行,每天每组进行一个实验,12个小组每天在不同的实验之间轮转(rotation),具体轮转方式见表5。采用该教学模式,每个实验只有4~6人同时参与,学生有充分的时间和机会了解原理、操作仪器、沟通交流,同时助教也可以随时观察学生表现、指导实验操作,及时指出问题并解答学生的疑问。该教学模式极大地调动了学生的学习热情,提高了学生在实验中的参与度,增强了学生的探索精神,减轻了仪器、试剂分配的压力,教学效果远超预期,学生反馈良好。

**表5:** 轮转机制表

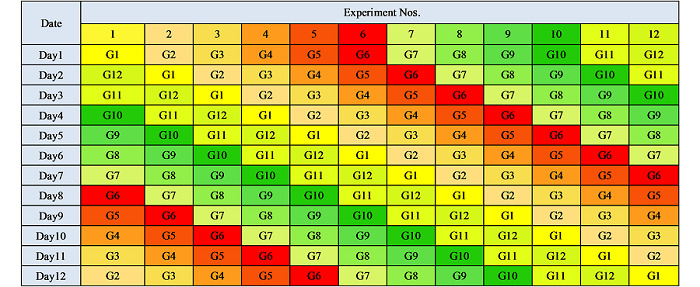

G: group.

### 3.3 大型仪器的利用

现代药物分析中仪器分析技术发展迅猛,对药学人才培养提出了更高的要求。培养熟练掌握大型仪器使用的药学专业人才,满足社会和行业的发展,是高校义不容辞的责任。本教学探索依托学院大型仪器平台,将HPLC、IR等教学仪器进行统一管理维护,同时调用平台上其他仪器设备参与实验教学,为学生提供了接触使用大型精密仪器的机会,具体仪器设备见表6。

**表6:** 大型仪器列表

Instrument	Model	Number	Number of experiments involved	Average time per student/h
Polarimeter	Anton Paar, MCP 200	1	1	1
Ultraviolet spectrophotometer	Agilent, Cary 60	9	3	4
Fourier transform infrared spectrometer	Bruker, TENSOR 27	1	1	1
HPLC	Agilent, 1220/1260	2	2	4
Liquid chromatograph-mass spectrometer	Waters, Acquity RDa	1	1	1
UPLC	Bruker, micrOTOF-Q II	1	1	1
NMR spectrometer	Bruker, AVANCE III, 600 MHz	1	extension	0.2

HPLC是药物分析领域最具优势、最常用的分离分析方法之一,因此本实验教学设置了3个涉及HPLC使用的实验,调用两台安捷伦HPLC仪和两台液相色谱-质谱联用仪,人均使用时长超过6 h,充分保证学生全面详尽地了解HPLC的工作原理、系统操作和数据分析。在阿司匹林与蒿甲醚等有关物质的杂质限量检查实验中,学生可利用液相色谱-质谱联用仪进一步对分离的杂质进行质谱分析。在使用傅里叶红外光谱仪的实验中,学生可近距离了解设备构造、体验压片等样品制备方法、解析谱图分析实验结果。学生对大型仪器的接触使用率达到100%,充分调动了学生的主观能动性,激发了学生的实验热情与探索激情,使实验教学更加深入全面、高质高效。

### 3.4 教学内容与其他课程衔接

药物分析是药学专业的核心课程,它上承有机化学、分析化学等基础课程,下启药物化学、天然药物化学等专业课程,其实验课程的教学内容与药物化学实验课程和天然药物化学实验课程联系也十分紧密。药物分析实验教学涉及两类药物——阿司匹林和蒿甲醚及其衍生物。在药物化学实验中,要求学生通过水杨酸合成阿司匹林,通过双氢青蒿素合成蒿甲醚,通过不断优化实验条件制备出3批符合《中国药典》标准的产品,在此过程中需要利用药物分析的各种分析检测方法,对合成产物进行过程监测,加深学生们对药品质量管控的认识。天然产物实验教学设计了从天然植物中提取分离青蒿素、双氢青蒿素、蒿甲醚等主要物质,药物分析实验教学中涉及的各种分析方法可再次得到应用。通过跟其他学科的紧密联系,强化了学生对药物分析方法的理解和掌握,打破了学科间的壁垒,有助于复合型人才的培养。

### 3.5 课后交流与反馈

每日实验结束后,助教根据本组学生的实验表现进行打分,学生以小组为单位上交实验报告。报告内容包括实验流程、实验结果、数据分析、存在的问题等,由助教进行批改。每周召开一次实验讨论会,学生分享展示本组的实验结果、提出实验过程中发现的问题、对其他小组的实验提出意见和建议;教师进行答疑,总结问题、内容归纳、数据分析。

全部实验课程结束后进行最终考核,该课程的最终考核并不停留在理论层面,而是设置A、B、C、D 4个实验,每个实验小组抽签决定考核的实验内容,直接考察学生的动手能力。教师根据学生的平时表现、实验报告、最终考核进行赋分,综合以上得到实验课成绩,这样的综合评价能够实现教学全过程质量控制和有效评价。

## 4 结论与展望

综上所述,在药物分析实验教学过程中,我们自2017年开始了助教辅助分组轮转式的实验教学探索,该模式引入“助教”“轮转”等教学方式,全面系统地设计了实验内容,贴近科研前沿和社会发展需求,强化课程间的紧密联系,并优化了考核方式,创建了更加符合教育规律、更有利于创新人才培养的实验教学模式。经过多年的不断尝试与探索,该模式日臻成熟完善,得到了学生和学院的一致好评,希望为国内高校实验课程的开展提供新思路。

今后,我们将与时俱进,不断落实“两性一度”,使药物分析的实验教学适应新时期药物分析的发展要求,培养全面的创新型药学卓越人才。
